# SEEG assistant: a 3DSlicer extension to support epilepsy surgery

**DOI:** 10.1186/s12859-017-1545-8

**Published:** 2017-02-23

**Authors:** Massimo Narizzano, Gabriele Arnulfo, Serena Ricci, Benedetta Toselli, Martin Tisdall, Andrea Canessa, Marco Massimo Fato, Francesco Cardinale

**Affiliations:** 1 Department of Informatics, Bioengineering Robotics and System engineering (DIBRIS), University of Genoa, Viale Causa 13, Genova, 16143 Italy; 2 Departement of Physiology Pharmacology and Neuroscience, CUNY Medical School, New York, New York USA; 3 Great Ormond Street Hospital for Children NHS Foundation Trust, Great Ormond St, WC1N 3JH, London, UK; 4 “Claudio Munari” Center for Epilepsy Surgery, Niguarda Hospital, Milan, Italy

**Keywords:** Epilepsy, SEEG, Automatic segmentation, Epileptic zone detections, GMPI, Medical imaging

## Abstract

**Background:**

In the evaluation of Stereo-Electroencephalography (SEEG) signals, the physicist’s workflow involves several operations, including determining the position of individual electrode contacts in terms of both relationship to grey or white matter and location in specific brain regions. These operations are (i) generally carried out manually by experts with limited computer support, (ii) hugely time consuming, and (iii) often inaccurate, incomplete, and prone to errors.

**Results:**

In this paper we present SEEG Assistant, a set of tools integrated in a single 3DSlicer extension, which aims to assist neurosurgeons in the analysis of post-implant structural data and hence aid the neurophysiologist in the interpretation of SEEG data. SEEG Assistant consists of (i) a module to localize the electrode contact positions using imaging data from a thresholded post-implant CT, (ii) a module to determine the most probable cerebral location of the recorded activity, and (iii) a module to compute the Grey Matter Proximity Index, i.e. the distance of each contact from the cerebral cortex, in order to discriminate between white and grey matter location of contacts. Finally, exploiting 3DSlicer capabilities, SEEG Assistant offers a Graphical User Interface that simplifies the interaction between the user and the tools. SEEG Assistant has been tested on 40 patients segmenting 555 electrodes, and it has been used to identify the neuroanatomical loci and to compute the distance to the nearest cerebral cortex for 9626 contacts. We also performed manual segmentation and compared the results between the proposed tool and gold-standard clinical practice. As a result, the use of SEEG Assistant decreases the post implant processing time by more than 2 orders of magnitude, improves the quality of results and decreases, if not eliminates, errors in post implant processing.

**Conclusions:**

The SEEG Assistant Framework for the first time supports physicists by providing a set of open-source tools for post-implant processing of SEEG data. Furthermore, SEEG Assistant has been integrated into 3D Slicer, a software platform for the analysis and visualization of medical images, overcoming limitations of command-line tools.

## Background

Up to 30% of patients with epilepsy are resistant to anti-epileptic drugs (AEDs) [1]. A subset of these patients suffers from partial epilepsy. These patients are potential candidates for the surgical removal of the epileptogenic zone (EZ), first defined as “the site of the beginning and primary organization of the epileptic seizures” [2, 3]. In order to characterize the EZ, clinical history and examination, medical imaging (e.g. Magnetic Resonance Imaging [MRI]), scalp EEG, and neuropsychological data are combined in order to derive a coherent hypothesis [4–7]. When non-invasive data are not sufficient, intracranial recordings may be used to define the EZ. On average, 25 to 30% of patients suffering from partial epilepsy are candidates for intracranial investigations (see. [8] and reference therein). Stereo-Electroencephalography (SEEG) is a methodology developed by Talairach and Bancaud at Hôpital Sainte Anne, Paris [3, 9, 10]. This methodology aims to record local field potentials through stereotactically implanted intracerebral electrodes. During the post-implant long-term Video-SEEG monitoring, epileptologists analyze the signals recorded from within the brain and attempt to define the EZ and a possible surgical resection plan. Therefore, the correct anatomical localization of all the implanted contacts is mandatory in order to define where the EEG traces are recorded from and also to facilitate radio-frequency thermal brain lesions performed through SEEG electrodes as a treatment option [11]. Electrode contact segmentation is a time-consuming task, as it often involves the localization of 100 to 200 contacts per patient. This task is usually carried out manually, by visual inspection of MRI or Computed Tomography (CT) scans [12] or using post-implant 2D photographs [13]; it is often incomplete (the majority of the contacts are not segmented) and can be inaccurate e.g. in cases of non-planar trajectories where artefacts may merge neighboring contacts into a single group of indistinguishable voxels. In recent years, several techniques for the localization of intracranial EEG electrodes have been proposed [14–18]. Most of the approaches aim to simplify the manual extraction of contact coordinates for grid/strips or for Deep Brain Stimulation (DBS) electrodes by fusing opportunely thresholded post-implant datasets with pre-implant MRIs [19, 20]. However, these approaches are not suitable for fully automatic localization of SEEG electrode contacts in complex arrangements (i.e. multiple electrodes per patients and converging trajectories). Mostly because visual extracting channel positions with respect to anatomical regions requires the visual integration of several imaging modalities and the manual selection the segmented contact positions. This approach is only suitable for a limited amount of channels as in the case of DBS electrodes (max two electrodes per patients with up to four recording/stimulating contacts). Recently, a novel algorithm has been proposed which has been specifically designed to segment SEEG contacts from a thresholded post-implant CT volume [21]. This algorithm has been implemented in an open-source tool called DEETO [22]. Despite its robustness, the tool has several limitations and lacks some advanced features: for example, it has no graphical interface, the type of multilead electrode (and thus the number of contacts) cannot be changed, and the parameter settings, which may be needed for complex cases such as touching electrodes, are only accessible to computer-expert users by modifying the code.

Moreover, visual inspection of post-implant data (MRIs, CTs or both) and acquired electrophysiological signals is routinely carried out to discriminate between contacts recording from grey or white matter, and to define the most probable neuronal source of the recorded activity.

In clinical and research settings, SEEG time-series are usually analysed using a bipolar (BP) referencing scheme, where each contact is referenced to its neighbour. Such approach might significantly distort the signal when bipolar contacts depict activity from neighbouring but functionally distinct areas, e.g. anterior and posterior banks of the central sulcus [23].

Recently it has been suggested that it may be more beneficial to use the nearby white matter contacts as ‘silent’ references to electrodes in grey matter [23]. This approach has been proven to alleviate the signal phase and amplitude distortion problems inherent in bipolar recordings and has allowed the characterization of phase and amplitude correlations [23] and long-range temporal correlations in the human brain with spatial and temporal patterns similar to those previously reported in Magneto-ElectroEncephalograhy (M/EEG) [24]. Taking advantage of a sub-millimetre resolution of lead localization process, in [23] authors formulated the Grey-Matter Proximity Index (GMPI) which represents the distance of a given contact from the white/grey boundary and therefore discriminates between contacts recording from white or grey matter (see GMPI estimator). The automatic computation of the GMPI requires ad-hoc scripts/tools that either are not freely available or need significant manual intervention in order to be included in standard clinical workflows.

On the other hand, post-implant images are usually fused with pre-implant MRI in order to visually estimate the position of each contact relative to the cerebral cortex and to derive its most probable neuronal source. All these operations require the integration of many different computer assisted tools (e.g., coregistration and visualization tools such as FSL/FLIRT [25]), and require significant knowledge and effort from the clinicians involved. To the best of our knowledge, such an integrated framework for post-implant processing does not exist. In this paper we present SEEG Assistant, a collection of tools that aim to assist neurosurgeons with the SEEG workflow, focusing on the post-implant phase.

## Implementation

**Fig. 1:**
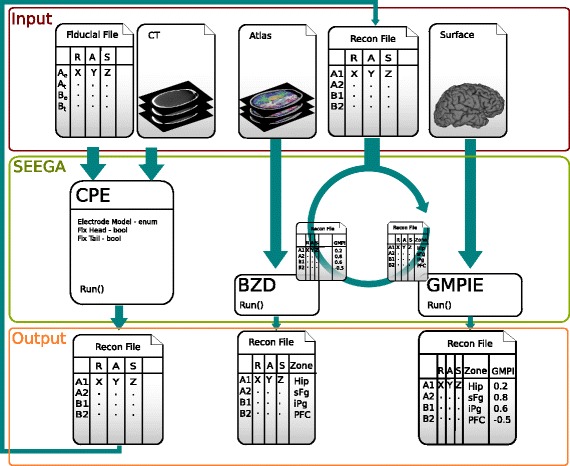
SEEG Assistant Framework is composed of three interconnected elements. The figure illustrates the *three modules* constituting the backbone of SEEGA module (*light green box*), their relations (*dark green arrow*), and I/O. Contact Position Estimator (CPE) extracts the contact positions from post-implant CT data provided the entry/target points for each implanted electrode (Fiducial File) and several parameters. These latter consist in the implanted electrode model (i.e., number contacts and inter-contact distance) and two boolean parameters. CPE outputs a fiducial file (Recon File) containing all segemented channel positions in the reference image (i.e., CT scanner space) along with contact label. In the test settings, electrodes are marked with a *single capital letter* (e.g., A or B) and channels are identified with increasing numbers (e.g., A1). The *next two modules* namely Grey Matter Proximity Index Estimator (GMPIE) and Brain Zone Detector (BZD) can be used without a specific order since they do not depends on each other. The former uses the segmented channel positions and *pial/white matter Surfaces* (e.g. Freesurfer) and computes for each contact the distance from *white/grey boundary* normalized to the *cortical thickness*. The latter uses contact positions and a volumetric probabilitic parcellation (e.g. Destrieux) and estimates the probability (i.e., proximity) of given source/anatomical area to be the generator of the recorded electrophysiological activity. All these information are added to the Recon File that can be saved for later usage or inspected from 3DSlicer interface

In this work we designed and implemented three modules, a graphical user interface (GUI) for each module, and integrated them in a 3DSlicer extension. Each module solves a specific task (Fig. 1). The first module, Contact Position Estimator (CPE), estimates the coordinates of the electrode contacts relative to the patient’s geometrical space by automatically detecting contact positions from a thresholded post-implant CT volume. The second module, the Brain Zone Detector (BZD), localizes the neuronal source of the recorded activity by automatically determining each contact position within a volumetric brain atlas, e.g. Destrieux [26]. The third one, the Grey Matter Proximity Index Estimator (GMPIE), enables automatic classification of white and grey matter contacts by automatically computing their GMPI indices.

SEEG Assistant is developed as an extension for 3D Slicer [27], a free and open-source software at the cutting edge of medical image analysis. It is mainly implemented in python (v.2.7) using VTK (v5.6) [28, 29] and QT libraries [30]. We decided to develop our tools as an extension of an existing software to exploit the vast collection of pre-processing tools available within 3D Slicer. This approach leads two benefits. First it does not link the users to a pre-defined methodology but grants some degree of freedom. Secondly, by addressing such specific tasks, it allows a more easy integration in existing manual/automated analysis pipeline in established clinical settings.

### Contact position estimator

The Contact Position Estimator segments the contact positions of each SEEG electrode in each subject’s geometrical space, requiring two inputs (Fig. 1). The first input is a pre-processed post-implant CT volume where multilead electrodes are imaged. The preliminary processing of this dataset includes two steps and should be performed outside the proposed tool. The former aims to remove the skull, as its voxel intensities lie in the same range as contacts, by subtracting a co-registered (affine registration, mutual information, performed using FSL/FLIRT [25]) pre-implant CT. The latter uses a manually selected threshold of voxel intensities to enhance boundaries between contacts and the background. All voxels above the selected threshold retain their original intensities while the remaining ones are fixed to zero. The second input is the Markups Fiducial (MF) List, a file that contains the planned entry and target points, defined as the most proximal and most distal points, with respect to the cortical surface, of each electrode, respectively. The file contains several lines, and each line may contain two different types of information: (1) comments (lines starting with #), and (2) a labeled point in the 3D space with the following format:

1 $$ l, x, y, z  $$

where *l* represents the unique electrode name and (*x, y, z*) the coordinates in millimeters of either the target or entry point referenced to the patient’s geometrical space. This file can be provided using coregistered pre-implant surgical coordinates or constructed directly using 3DSlicer tool (i.e., Markup module). CPE reads this information from 3DSlicer memory and assumes that both the CT scan and the MF file refer to the same geometrical space. From the MF list, the module constructs a set of electrodes coupling their entry and target points using electrode label (e.g. A):

2 $$ L_{i}=< E_{i},T_{i} >$$

where *E*_*i*_ is the entry point and *T*_*i*_ is the target point of the electrode labeled *L*_*i*_. Supported naming strategies are (1) the same capital letter or (2) capital letter and capital letter followed by a number (e.g. A1) for entry and target points, respectively. Afterwards, the module creates an interactive table where the user can choose, for each electrode, the manufacturer model used in the implant. The default configuration assumes that the patients have been implanted with semi-rigid SEEG electrodes manufactured by DIXI Medical (Microdeep Intracerebral Electrodes - D08®, Depth Electrodes Range 2069®). Many models of electrodes exist, with varying lengths and numbers of contacts. However, we provide a configuration file where different electrode settings can be added without the need to change the code. For each electrode the User Interface (UI) also displays two check boxes: Tip and Cortex. These are the geometrical points used to estimate the final axis. The Tip is the most distal point on the tip of the electrode, while the Cortex is the point closest to the cortical surface. Tip and Cortex are usually computed starting from the Target and Entry point of the MF file, respectively. Thus, when Cortex (Tip) box is checked, the CPE does not estimate its relative point but assumes the line connecting the unchecked (that is, estimated) and checked (that is, taken as is) as the electrode axis. If a user checks one (or both) of these boxes, he should also be sure that the Targets/Entries of the MF list are as accurate as possible with respect to the Tip/Cortex positions. 3DSlicer visualizes points contained in MF list as spheres in the 3D visualization. Thus, from the interface it is possible to manually adjust every point contained in the list or even add or remove any of them.

Finally, once all settings have been fine tuned, the user actively triggers the computation by pressing the Start Segmentation button and, for each selected electrode from the MF list, the module executes an external (to 3DSlicer) tool called deetoS [31]. This tool has been implemented in C++ exploiting advanced features of the ITK libraries [32, 33] which, at the time of writing the code, were not fully ported in python nor included in standard 3DSlicer installation package. For this reason, deetoS has been called as external tool within the UI. deetoS is an open-source software that has been constructed as a branch of the tool DEETO [22] to ease the integration with 3DSlicer. More precisely, we simplified the command line, by allowing the segmentation of one electrode at a time (DEETO reads the MF and processes the entire implant at in one step), but also including the option to set the type of electrode used for the segmentation. In this section we will give a general overview of the algorithm; for further detail please refer to [21].

**Fig. 2:**
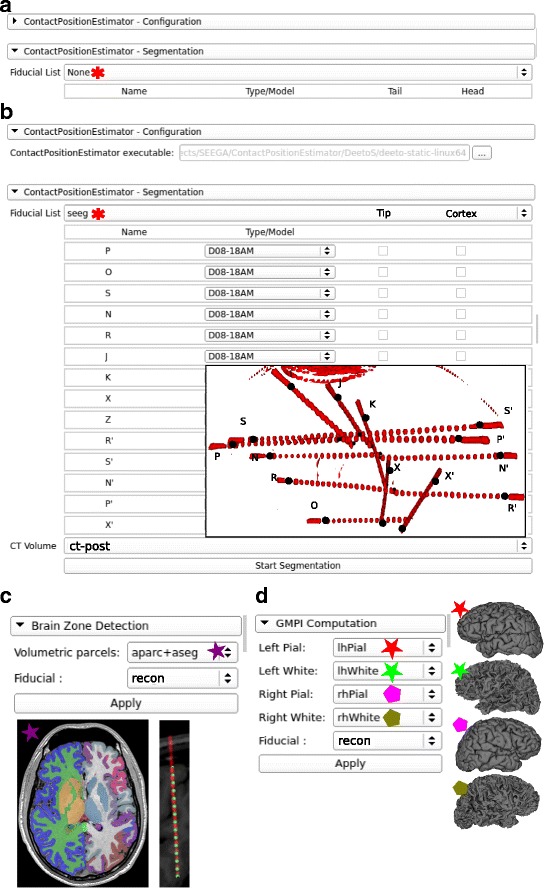
Interfaces and workflow of SEEGA. This figure shows the interfaces designed to interact with the underlying algorithms and provides an example of the workflow. All *drop-down listboxes* are populated with data included in the 3DSlicer scene. **a** Initial configuration panel of CPE where a fiducial file needs to be selected. **b** Shows the path of the deetoS binary file provided within SEEGA installation. Once a fiducial file (*seeg*) has been selected a table is populated with recognized electrodes with valid entry/target pairs. The *drop-down list* next to the electrode label is used to select its specific model which univocally defines number of contacts and inter-contact distances. An example of what is shown in the *3D View panel* wihtin 3DSlicer is provided as inset with post-implant thresholded-CT meshes (ct-post - *red*) and fiducial file shown as markups (*black dots*) with letters representing each electrode. **c** Shows the BZD interface where both BZD takes a volumetric parcellation (aparc+aseg - *purple star*) and the recontructed data (recon). Examples of such inputs are shown below the interface. **d** Shows GMPIE interface where the five inputs are defined as *left* (*red*) *right* (*green*) *pial* (*star*) and *white* (*pentagon*) surfaces together with segmented channel positions (recon)

**Fig. 3:**
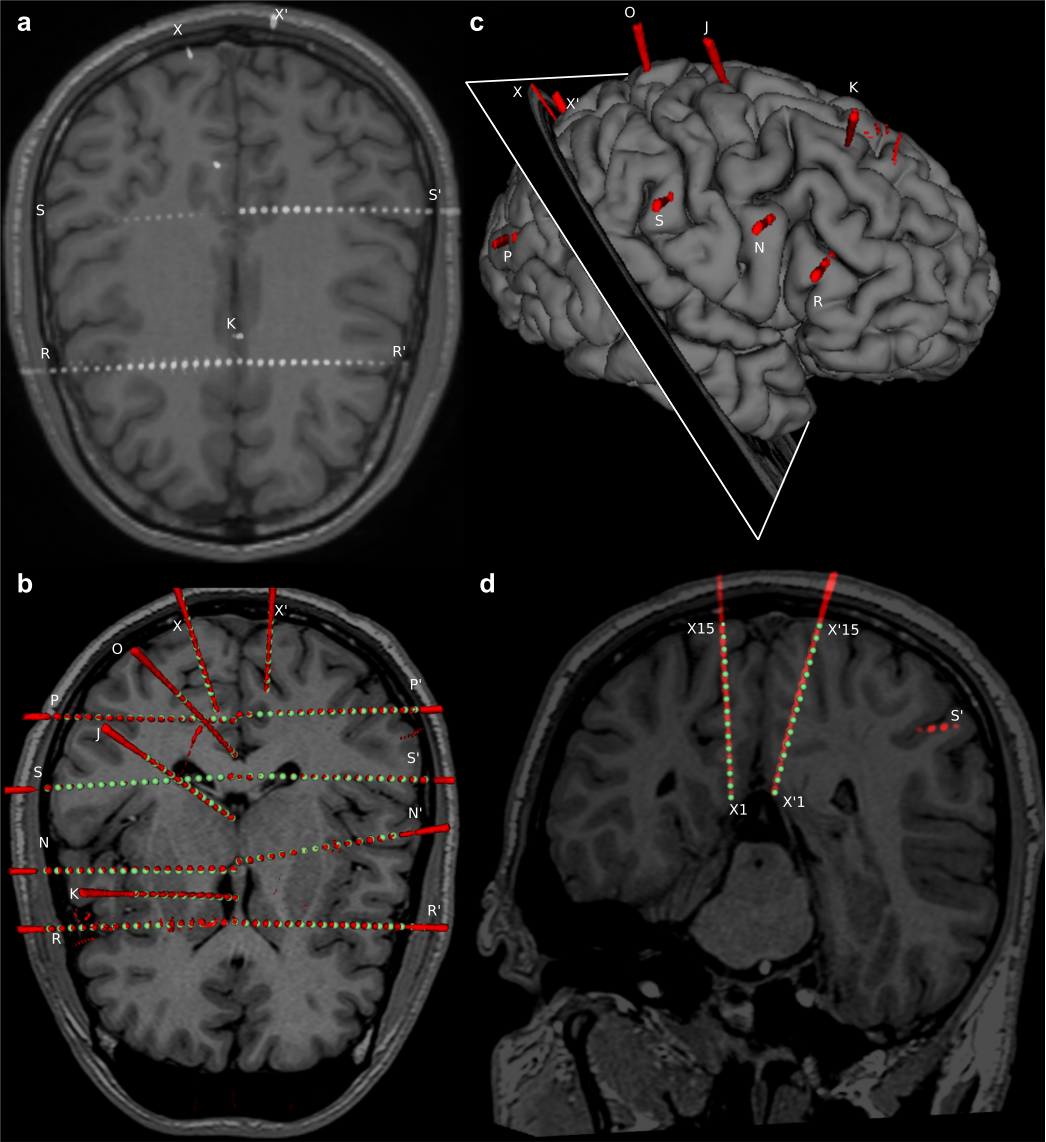
CPE out performs manual segmentation in complex and critical cases. **a** As an example of SEEG complexity, we show MRI and thresholded post-implant CT scans for one subject from our cohort. Contacts are shown as groups of *white voxels*. This case illustrates the complexity of SEEG implants with electrode shafts following non-planar directions (e.g. X), shafts targeting almost the same geometrical point (e.g. R and R’). **b** CPE segments all contacts (*green spheres*) belonging to each electrode from post-implant CT scans, represented here as *red 3D meshes* obtained tesselleting the thresholded data to ease visualization. **c** Show the right pial surface with 3D post-implant thresholded-CT meshes and the *cut plane* used in panel **d** where the example of X and X’ electrodes are shown. Those examples represent the case of non-planar insertion trajectories which yielded an artefactually fused electrode. CPE integrating the knowledge of the electrode model can segment the contact positions more accurately than visual inspection

DeetoS takes three parameters as input: (i) the CT volume, (ii) the coordinates of two points, (iii) the parameters describing the electrode type (i.e., inter-contact distance and number of contacts). It then outputs a set of coordinates representing the centre of each segmented contact and saves them as markup entries along with a label formed by a unique capital letter for each electrode followed by an integer. The segmented points are then ordered alphabetically in the list, from the deepest (e.g., A1) to the most external point (e.g., A18) with respect to the cortical surface. The algorithm executes two main steps: *electrode axis estimation* and *electrode contact segmentation*. The electrode axis estimation relies on the knowledge of two initial points, namely the entry point and the target point, defining the planned electrode axis. The electrode axis estimation computes the final electrode axis, i.e. a line joining the cortex and tip points. The cortex point estimation is usually executed by computing the centre of mass of a region around the entry point. In a real implant, the electrode entries are far from each other, while the tips can be very close: for this reason the algorithm used to compute the cortex cannot be used to estimate the tip. Indeed, the tip is usually estimated starting from the cortical entry point of the electrode and progressively computing the centre of mass of the points lying on the axis defined by the entry and target points. Once cortex and tip points have been estimated, deetoS proceeds with the electrode contact segmentation step. It iteratively locates each contact within a geometrical-constrained search space on the estimated axis. The search-space is defined by two strong constraints: the former represents the fixed inter-contact distance (i.e. the distance between two subsequent contacts), while the latter states that the axis deviation should be less than 10 degrees, since those deviations can only occur within electrode cables connecting two adjacent contacts. The contact centroids are then approximated with the centre of mass of the radiological artefacts that satisfies all the strong constraints (Fig. 2 c). Occasionally, in some rare and complex cases with default configuration - i.e. standard electrode model and planned entry/target points, the CPE module fails to correctly segment some electrodes. Simple visual inspection of the 3D scene allows identification of these cases (Fig. 3). These errors only arise during axis estimation, and this is most frequently due to proximity with other electrodes. Neighbouring electrodes can influence the centre of mass computation applied in the estimation of the real axis, since the algorithm relaxes the geometrical constraints to find a suitable solution. On the other hand, once the axis has been estimated, the search for the contact positions restores these constraints and the solution cannot diverge as the algorithm proceeds along the estimated line. Thus, when the estimated axis diverges from the real one, it is possible to set it manually by checking the tip and cortex boxes in the electrode configuration table. If a check box is active, deetoS does not compute the corresponding point, and thus the entry and target points represent the electrode axis.

### Brain zone locator

The Brain Zone Locator module locates each contact with respect to the brain region it occupies. The module uses a pre-computed volumetric representation of a probabilistic atlas, such as the Destrieux [26] or Desikan-Killiany atlases [34] included in Freesurfer. It takes two inputs: (1) a volumetric parcellation co-registered with subject geometrical space, where each voxel has been tagged (automatically or manually) with a numeric tag representing the most probable region within which it is located, and (2) a set of points representing lead positions. For each contact, it constructs a spherical Region Of Interest (ROI) with 7 mm diameter centered in its centroid and extracts the fraction of voxels representing a given area contained within the ROI. We chose 7 mm as diameter because contact centroids are 3.5 mm apart. Thus, 3.5 mm on both sides of the volume ensure a partial overlap between adjacent contacts. Of note, different electrode settings might require different side lengths. Thus the parameter can be manually changed by the user through the interface. These values, together with the region name, are presented with the MF description and saved along with lead positions. The module assumes that the volume and lead positions lie in the same geometrical space.

### GMPI estimator

To enable automatic classification between white and grey matter contacts, we included the Grey Matter Proximity Index Estimation for each segmented contact. The module takes as input a set of labelled points and the meshes describing the pial and white surfaces for both hemispheres. Theoretically, one investigator can use any tool that provides a brain segmentation and which outputs 3D meshes for grey and white matter boundaries. In this work we estimated cortical pial and white surfaces by means of Freesurfer tools [35] using standard 3D T1 FFE MRIs data and default settings (i.e. recon-all). For detail about the usage of Freesurfer tools, advanced parameter settings, and complete description of outputs please refer to the relevant documentation [35]. It should be noted that Freesurfer surfaces are natively represented in a centered geometric space. Thus, a simple translation is required to align the center of scanner space, where contacts are represented, to surface space.

The GMPI is defined as the distance between the contact position and the nearest vertex of the white-grey surface, scaled by the cortical thickness at that point. Thickness is here estimated as the distance between the nearest pial and white-matter-grey-matter interface (WMGMI) vertices along the normal axis to the cortex. This measure has been proven to be reliable with respect to real cortical thickness assessed on post-surgical histological investigation of resected tissue [36, 37]. GMPI can be mathematically formalised as follows:

3 $$ GMPI = \frac{(C - W) \cdot (P-W)}{|P-W|}  $$

where *P*(*x,y,z*),*W*(*x,y,z*) and *C*(*x,y,z*) are the pial vertex, WMGMI vertex and contact coordinates, respectively. GMPI is an unbounded measure that can be used to automatically classify grey and white matter contact. GMPI values between zero and one indicate that the contact midpoint is located within grey matter, whereas negative values indicate that the contact midpoint is in white matter. However, electrical fields generated in grey matter spread also to nearby white matter. Moreover, the electrode contact physically spans a 2 mm long volume with a radius of 0.4 mm around the midpoint. Thus, in [23] authors suggest to use −0.3 as the GMPI threshold for grey/white matter determination. They have also demonstrated that with this threshold none of the white matter contacts are misclassified as grey matter contacts. It should be noted that, using this formulation GMPI values relative to subcortical leads are invalid. This is due to the normalization factor (that is the cortical thickness). Using two sides of a given subcortical structure (e.g. lateral and ventral portion of hippocampus) is strongly dependent on the insertion direction of the lead. Two electrodes coming from different but converging directions to the same structures (e.g. hippocampus) might yield different GMPI values because of the different cutting planes. We thus decided to include the labelling of contacts in volumetric parcellation, rather than simply using surface based atlases, to automatically classify pure white matter contacts that may be used as silent references for subcortical structures. These are defined as the contacts that are surrounded by only white matter fibers in the ROI used in the Brain Zone Detector.

### User interface design

Each module contained in SEEGA has its own UI that allows user interaction with the implemented algorithms and the definition of parameters (if any). Since all modules have been implemented as 3DSlicer extensions, the GUI for each module (Fig. 2) is based on the Qt library. The CPE Interface is divided in two parts (Fig. 2 a). The former, CPE-Configuration, allows the definition of the deetoS path, while the latter, CPE-Segmentation, consists of two drop-down lists for the selection of the appropriate inputs. Once the fiducial has been chosen, a table is produced (Fig. 2 b), where the user can select, for each electrode, the Type/Model and fix the Cortex/Tip points. The last drop-down list allows the user to choose from the CT volumes loaded in Slicer. Both the Brain Zone Detection (Fig. 2 c) and the GMPI Computation Interfaces (Fig. 2 d) provide several drop-down lists, depending on the number of mandatory inputs, and the button that triggers the algorithm execution.

### Modules validation

In order to test the overall quality of SEEG Assistant, we used the three modules to process 40 SEEG implants comprising 555 electrodes and 9626 contacts. We also performed manual segmentation, as routinely performed in the clinical setting in a subset of 8 patients (98 electrode and 1302 contacts). These implants were performed at “Claudio Munari” Centre for Epilepsy Surgery. The methodology routinely adopted to implant SEEG electrodes and to post-process neuroimaging datasets has been described previously [8, 38]. We initially ran the CPE module using the surgically planned entry and target points and default electrode models (i.e. 18 contacts 1.5 mm inter-contact distance). We confirmed correct alignment of contact centroids with respect to the post-implant imaging dataset (Fig. 3). To do this, we visualized the estimated contact centroids as spheres (i.e. markups) of 2 mm diameter in the 3D view along with 3D meshes representing radiological artefact surrounding each contact (see Fig. 3 b and d). If all markups were contained within the corresponding artefacts we defined those contacts as being correctly segmented. On the other hand, we defined a contact as being incorrectly segmented if the corresponding centroid was estimated outside of the reconstructed radiological artefact or was not lying on the same axis as its neighbours.

To quantitatively evaluate the CPE accuracy, a gold standard for contact positioning must be defined, and the distance between real and estimated coordinates computed. However, in the clinical setting the position is visually assessed on post-implant imaging. This cumbersome task might lead to errors, in particular when electrodes are implanted along the cranio-caudal axis. Furthermore, the resolution of our CT scanner is lower along the z axis, thus the radiological artefacts for each contact may become merged together (e.g. Fig. 3 c and d, electrodes X and X’). Thus, computing euclidean distance between estimated and visually assessed channel positions may be inaccurate. For these reasons, we assessed CPE accuracy with two metrics based on the known physical properties of the implanted electrodes. We computed the distance of each contact from its estimated axis ($D^{a}_{ax},D^{m}_{ax}$) and the mean inter-contact distances for each electrode ($D^{a}_{ic},D^{m}_{ic}$) using automatic (*D* ^*a*^) and manual (*D* ^*m*^) methods. These two measures give a quantitative overview of the concordance between the reconstructed and the real electrode geometries. The former should be close to 0 mm since contacts should all lie on the same axis. In this case, we estimated the electrode axis by means of a linear interpolation of the contact positions, since the real axis is neither known nor exactly computable from just post-implant non-invasive imaging data. The second measure should be 3.5 *mm* for most commonly used electrode models.

## Results

In this work we present SEEG Assistant, a 3DSlicer extension designed to assist neurosurgeons in post-implant image processing of SEEG implants. The proposed extension is composed of three modules that aim to (i) localize the position, (ii) estimate the most probable cortical sources of the recorded signals, and (iii) extract GMPI and volumetric labelling to enable later classification of white-grey matter contacts.

### Visual validation

We assessed contact position accuracy by visual investigation of segmented marker points overlayed on CT post-implant datasets (see Modules validation). The CPE module produced correct segmentation in 87.57% (i.e. 8429 out of 9626 contacts considered) of the cases with default settings only, i.e. using pre-implant information and no-corrections or fixed parameters. In the remaining (1197), more complex cases, the CPE results required some manual corrections. For example, in cases where electrodes were in close proximity (e.g. Fig. 3 b), the segmentation process required manual verification of the correct target point position (i.e. the tip of the electrode) and fixing the tip point by checking the corresponding box. Using this approach among the remaining leads, 78.27% (i.e. 937 out of 1197) of the cases were correctly localized by checking cortex/tip or both, while in 17.39% (i.e. 203 out of 1197) of cases the target point or the entry point were missing from the MF file.

### Quantitative analysis

**Fig. 4:**
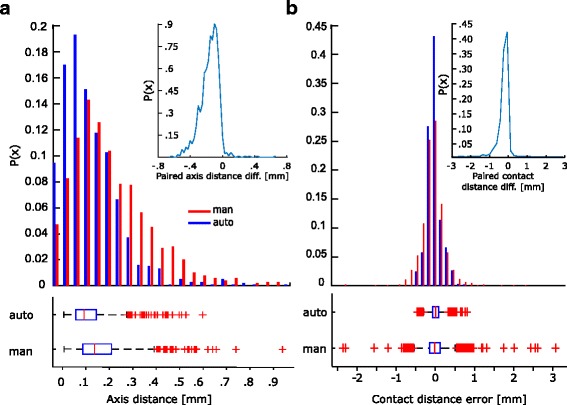
CPE module provides more accurate results compared to manual segmentation. **a** Contact distances from axis are on average similar between manual (*blue*) and automatic (*red*) segmentation. In general automatic segmentation performs better in keeping all contacts aligned to their axis. Probability distribution shows that the paired differences of automatical and manual defined contact to their axis is signficantly (*p* < 0.05, paired wilcoxon test) smaller than zero. As expected, manual segmentation yields higher variability and a larger fraction of outliers. **b** Inter-Contact distributions show gaussian-like distributions. Probability distribution of paired difference of automatic and manually defined contacts shows that our method significantly (*p* < 0.05) out performs compared to the manual case in representing real inter-contact distance. Finally, automatic segmentation shows a smaller variability compared to manual segmentation

In the absence of a ground truth to test performance against, we tested whether automatically defined contact positions reconstruct real electrode geometry more accurately than manually segmented ones. We assessed this by defining two measures namely the distance from each contact to its electrode axis and the inter-contact distance (see Modules validation). We report that 95% of the automatically segmented contacts were less than 0.2 mm from their axes (Fig. 4 a). Conversely, manual segmentation yielded higher variability and several outliers ≥ 0.85 mm (Fig. 4 a). Moreover, we tested whether the difference between automatic and manual distances were significantly (*p*<0.05) smaller than zero ($P=\Pr (D^{a}_{ax} < D^{m}_{ax})$) by means of a one tailed paired wilcoxon test. We report that the difference between automatic and manual distances from axes is statistically significant.

The majority of the contacts in the automatic estimation are 3.5 *mm*±0.3 *mm* apart from each other. The variability in the manual segmentation case is greater (3.4 *mm*±0.6 *mm*), and has several outliers with an inter-contact distance of more than 6 *mm* (Fig. 4 b). The higher variability of the manual data could be easily explained by imprecision in discriminating channels in transverse electrodes. In fact, in these cases, radiological artefacts fuse neighbouring contacts as mentioned earlier. Also in this case, we assessed statistical significance using a paired wilcoxon test. We subtracted the expected mean value of 3.5 *mm* (i.e. physical mean distance among neighbouring channels) from both populations ($\hat {D}^{a}_{ic} = D^{a}_{ic}-3.5$) and ($\hat {D}^{m}_{ic}=D^{m}_{ic}-3.5$). We applied the same statistical approach as above, testing whether automatic method yields inter-contact distance closer to the expected value $P=\Pr (|\hat {D}^{a}_{ic}| < |\hat {D}^{m}_{ic}|)$. Also in this case, the inter-contact distances are significantly smaller (*p*<0.05, paired wilcoxon test) from those obtained manually.

Finally, the mean time necessary to complete an implant segmentation was 75±25 min and 15±5 s for manual and automatic segmentation, respectively. It is important to note that we did not record the time spent preparing the scenes (i.e. choosing and loading data) or pre-processing data (e.g. Freesufer pipeline) for each patient, since it is equivalent for both methodologies. Thus, the reported average times are relative only to the average time required to run SEEGA tools on a standard Linux workstation with i7-core and 8 GB RAM.

### Automatically localized implants accurately reflect their position relative to most probable pathological areas

**Fig. 5:**
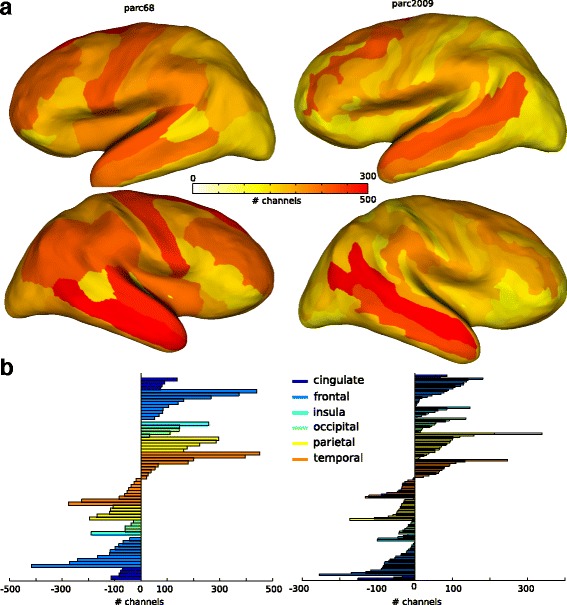
BZD estimates most probable electrical sources using volumetric atlases. **a** Number of contacts recording from each parcel is shown on *top* of the inflated surfaces of the cerebral cortex. *Color code* is shown in the *color bar* and represents the *less* (*white*) to *most* (*red*) sampled regions. Two atlases have been used to test the algorithm: Desikan-killeany (parc68 - *left column*) and Destrieux (parc2009 - *right column*). Both atlases yields a similar spatial distribution. The existing differences can be due to the different parcel resolutions. **b** Number of contacts from each parcel for both parc68 and parc2009 are shown as *color bar* histograms divided between cerebral lobes. Here similar patterns can be seen across atlases

Moreover, the Brain Zone Locator was tested with two different Atlases, namely Desikan-Killiany [34] and Destrieux [26]. These atlases have different spatial resolution making a direct comparison impractical. We computed the parcellation coverage as the number of recording contacts from each parcel. We represented these values on top of inflated surfaces and as histograms (Fig. 5). Using both atlases, it can be seen that the most frequently sampled regions are located in frontal and temporal regions which are known to be the major sites for focal epileptogenic zones [39] while posterior parts of the brain represent more rare cases [40].

### SEEG implants yield higher spatial sampling of grey compared to white matter

**Fig. 6:**
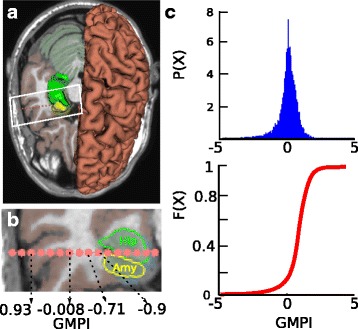
GMPI reflects channel position relative to cerebral cortex. **a** An MRI inset is shown with atlas (i.e., Destrieux) and three meshes are shown representing Hippocampus (*green*) Amygdala (*yellow*) and pial (*red*) surfaces. Segmented contact positions are represented as *pink spheres centered* in the estimated position. **b** This panel shows a *zoom-in* on the *segmented contact plane* where it can be seen the contact positions, the intersection between plane and subcortical structures (i.e., Hip and Amy). Cortical sheet has been marked with *brown color*. The *axis below* shows that GMPI decreases while the shaft penetrates *white matter fibers* with increasing distance from cortical sheet. **c** Probability (*top*) and cumulative (*bottom*) distributions of GMPI values across all cortical contacts

SEEG Assistant also estimates GMPI and volumetric labelling that can be used to determine which contacts are located in Grey/White matter and their associated brain regions. Generally, trajectory planning aims to position as many contact as possible in close proximity to pathological cortical and subcortical regions limiting the amount of contact recording from white matter (Fig. 6 a and b). We computed the distribution of GMPI values for all the cortical channels – i.e. excluding those targeting subcortical regions (Fig. 6 c). We report that the vast majority of cortical channels have GMPI greater than −0.3 which indicates that many channels are actually recording within (0<*x*<1) or in close proximity to (−0.3<*x*<=0) the cortical ribbon. Moreover, given the limitation of using GMPI only for cortical channels, in the current cohort the index is still valid in 97% of the cases. This index along with volumetric labeled contacts, are key features used in solving the issue of finding silent references for each cortical and subcortical channel. Of note, at the moment SEEG Assistant does not classify grey/white channels but instead estimates the parameters used to solve the classification problem.

## Discussion

We introduce a set of tools to aid the post-surgery assessment of SEEG implants. The provided tools are built as an extension called SEEGA and are integrated in 3D Slicer exploiting its advanced medical imaging processing capabilities. Our set of tools enrich the collection of extensions of 3D Slicer making it a perfect tool for surgical planning and post-surgery assessment. We preferred creating an extension to an existing software rather than a new stand-alone all-in-one tool, to allow users to define their preferred set of pre-processing steps. This ensures later extensibility and, we hope, a wider adoption of our software across centres rather than limiting the usability to the test centres.

The set of algorithms have been tested both qualitatively as well as quantitatively using a cohort of 40 and a subset of 8 SEEG implants, respectively. We show that the CPE module correctly reconstructs contact positions in native scanner space of each patient in the vast majority of the cases without any supervision. Our localization tool accurately segments contact positions with an error less than 0.5 *mm* [21] which is directly comparable to the image resolution of the testing CBCT scanner and in line with that reported elsewhere [19]. Of note, Hebb and colleagues reported a smaller localization error but used a standard CT scanner with higher resolution while we used a CBCT intra operative mobile scanner which has been proven to provide equivalent final accuracy [14].

We also demonstrate that automatically reconstructed contact positions accurately reflect real electrode geometry such as inter-contact distances and distance from estimated electrode axis. Moreover, these results are significantly more accurate than those manually defined even in complex cases.

This work adds significantly to the literature. Other works exist to automatically or semi-automatically assess post-implant localization in intra-cerebral electrode implants. The vast majority attempt to solve a similar problem assessing the localization of grids/strips electrodes [17, 18]. Only a few studies deal with Depth Electrodes and most are used in Deep Brain Stimulation [19]. In DBS, surgeons implants at most two electrodes targeting Subthalamic Nuclei (STN) in the therapy of Parkinson’s disease. Published method exploits the simplicity of intracerebral implantation to build a fully automated localization algorithm. This approach is not suitable for our cases due to the many (up to 20) multilead electrodes implanted during SEEG investigations.

Another work from Princich and colleagues [41] advances a methodological procedure to support post-implant leads labelling in SEEG. Their approach aims to ease and standardize the visual localization and identification of neighbouring anatomical brain structures. We advance their approach by enabling automatic localization and anatomical labelling using the same open-source set of toolboxes, namely Freesurfer and 3D Slicer. Indeed, we present a tool to automatically label each channel in respect to the most probable neuronal source using probabilistic atlases. Using such tool we show that the most frequently sampled brain areas are located in frontal and temporal regions which are known to be the major sites for focal epileptogenic foci [39], while posterior parts of the brain represent more rare cases [40].

## Conclusions

In this work we present SEEG Assistant, a piece of software specifically designed to support neurosurgeons in post-seeg implant processing. We exploit an open-source paradigm, easily accessible and integrated interface connected to 3DSlicer. Our tool significantly eases the SEEG post-implant analysis by drastically reducing the time required for segmentation and localization. Our analyses show that automatic segmentation is significantly more accurate than manually extracted coordinates. Furthermore, SEEG Assistant provides epileptologists with segmentation methods and brain region labeling, all included in an easily extensible and user-friendly interface. This tool may aid the clinical implementation of SEEG data, by facilitating the interpretation of recorded brain activity. Given the growing interest in SEEG methodology, in both the clinical environment and the neuroscience research community, we believe that this tool provides an interactive user interface able to sensibly reduce time in accurately assessing post-implant SEEG contact positioning relatively to neighbouring anatomical structures. Finally, this tool can be used on a large cohort of historical data to extract the most frequent trajectories. These in turn can be used for initialising the search space in an automatic planning software.

## Abbreviations

BP: Bipolar
BZD: Brain zone detector
CPE: Contact position estimator
CT: Computed tomography
EEG: Electroencephalography
EZ: Epileptogenic zone
GMPI: Grey-matter proximity index
GMPIE: Grey matter proximity index estimator
GUI: Graphical user interface
MF: Markups fiducial
MRI: Magnetic resonance imaging
ROI: Region of interest
SEEG: Stereo-electroencephalography
UI: User interface

## References

[1] Kwan P, Brodie MJ (2000). Early identification of refractory epilepsy. N Engl J Med.

[2] Munari C, Bancaud J, Porter RJ, Morselli PL (1985). The Epilepsies. The role of stereo-electroencephalography (SEEG) in the evaluation of partial epileptic seizures.

[3] Bancaud J, Talairach J, Bonis A, Schaub C, et al.La stéréo-électroencéphalographie dans l’épilepsie: informations neurophysiopathologiques apportées par l’investigation fonctionelle stéréotaxique: Paris Masson & Cie Editeurs; 1965.

[4] Munari C, Lo Russo G, Minotti L, Cardinale F, Tassi L, Kahane P, Francione S, Hoffmann D, Benabid AL (1999). Presurgical strategies and epilepsy surgery in children: comparison of literature and personal experiences. Childs Nerv Syst.

[5] Cossu M, Cardinale F, Castana L, Citterio A, Francione S, Tassi L, Benabid AL, Lo Russo G (2005). Stereoelectroencephalography in the presurgical evaluation of focal epilepsy: a retrospective analysis of 215 procedures. Neurosurgery.

[6] Cossu M, Lo Russo G, Francione S, Mai R, Nobili L, Sartori I, Tassi L, Citterio A, Colombo N, Bramerio M, Galli C, Castana L, Cardinale F (2008). Epilepsy surgery in children: results and predictors of outcome on seizures. Epilepsia.

[7] Cossu M, Schiariti M, Francione S, Fuschillo D, Gozzo F, Nobili L, Cardinale F, Castana L, Russo GL (2012). Stereoelectroencephalography in the presurgical evaluation of focal epilepsy in infancy and early childhood. J Neurosurg Pediatr.

[8] Cardinale F, Cossu M, Castana L, Casaceli G, Schiariti MP, Miserocchi A, Fuschillo D, Moscato A, Caborni C, Arnulfo G, Lo Russo G (2013). Stereoelectroencephalography. Neurosurgery.

[9] Cardinale F, González-Martínez J, Lo Russo G (2016). SEEG, happy anniversary!. World Neurosurg.

[10] Cardinale F, Casaceli G, Raneri F, Miller J, Lo Russo G (2016). Implantation of stereoelectroencephalography electrodes. J Clin Neurophysiol.

[11] Cossu M, Fuschillo D, Cardinale F, Castana L, Francione S, Nobili L, Lo Russo G (2014). Stereo-EEG-guided radio-frequency thermocoagulations of epileptogenic grey-matter nodular heterotopy. J Neurol Neurosurg Psychiatry.

[12] Darcey TM, Roberts DW (2010). Technique for the localization of intracranially implanted electrodes. J Neurosurg.

[13] Dalal SS, Edwards E, Kirsch HE, Barbaro NM, Knight RT, Nagarajan SS (2008). Localization of neurosurgically implanted electrodes via photograph-MRI-radiograph coregistration. J Neurosci Methods.

[14] Lee DJ, Zwienenberg-Lee M, Seyal M, Shahlaie K (2015). Intraoperative computed tomography for intracranial electrode implantation surgery in medically refractory epilepsy. J Neurosurg.

[15] Stieglitz LH, Ayer C, Schindler K, Oertel MF, Wiest R, Pollo C (2014). Improved localization of implanted subdural electrode contacts on magnetic resonance imaging with an elastic image fusion algorithm in an invasive electroencephalography recording. Neurosurgery.

[16] Kovalev D, Spreer J, Honegger J, Zentner J, Schulze-Bonhage A, Huppertz HJ (2005). Rapid and fully automated visualization of subdural electrodes in the presurgical evaluation of epilepsy patients. AJNR Am J Neuroradiol.

[17] Hermes D, Miller KJ, Noordmans HJ, Vansteensel MJ, Ramsey NF (2010). Automated electrocorticographic electrode localization on individually rendered brain surfaces. J Neurosci Methods.

[18] Sebastiano F, Di Gennaro G, Esposito V, Picardi A, Morace R, Sparano A, Mascia A, Colonnese C, Cantore G, Quarato PP (2006). A rapid and reliable procedure to localize subdural electrodes in presurgical evaluation of patients with drug-resistant focal epilepsy. Clin Neurophysiol.

[19] Hebb AO, Miller KJ (2010). Semi-automatic stereotactic coordinate identification algorithm for routine localization of deep brain stimulation electrodes. J Neurosci Methods.

[20] van Rooijen BD, Backes WH, Schijns OEMG, Colon A, Hofman PAM (2013). Brain imaging in chronic epilepsy patients after depth electrode (stereoelectroencephalography) implantation. Neurosurgery.

[21] Arnulfo G, Narizzano M, Cardinale F, Fato MM, Palva JM (2015). Automatic segmentation of deep intracerebral electrodes in computed tomography scans. BMC Bioinforma.

[22] Seeg electroDE rEconstruction Tool (DEETO). http://github.com/mnarizzano/DEETO. Accessed 26 May 2016.

[23] Arnulfo G, Hirvonen J, Nobili L, Palva S, Palva JM (2015). Phase and amplitude correlations in resting-state activity in human stereotactical {EEG} recordings. NeuroImage.

[24] Zhigalov A, Arnulfo G, Nobili L, Palva S, Palva JM (2015). Relationship of fast- and slow-timescale neuronal dynamics in human MEG and SEEG. J Neurosci Off J Soc Neurosci.

[25] Jenkinson M, Bannister P, Brady M, Smith S (2002). Improved optimization for the robust and accurate linear registration and motion correction of brain images. NeuroImage.

[26] Destrieux C, Fischl B, Dale A, Halgren E (2010). Automatic parcellation of human cortical gyri and sulci using standard anatomical nomenclature. NeuroImage.

[27] Fedorov A, Beichel R, Kalpathy-Cramer J, Finet J, Fillion-Robin JC, Pujol S, Bauer C, Jennings D, Fennessy F, Sonka M, Buatti J, Aylward S, Miller JV, Pieper S, Kikinis R (2012). 3D Slicer as an image computing platform for the quantitative imaging network. Magn Reson Imaging.

[28] Schroeder W, Martin K, Lorensen B. The visualization toolkit: an object-oriented approach to 3D graphics, 4th edn: Kitware Inc.; 2003.

[29] The Visualization Toolkit Library. http://www.vtk.org. Accessed 26 May 2016.

[30] Qt Library. http://www.qt.io. Accessed 26 May 2016.

[31] deetoS (deeto for Slicer). http://github.com/mnarizzano/DEETO/tree/deeto-slicer. Accessed 26 May 2016.

[32] Yoo TS, Ackerman MJ, Lorensen WE, Schroeder W, Chalana V, Aylward S, Metaxas D, Whitaker R (2002). Engineering and algorithm design for an image processing Api: a technical report on ITK–the insight toolkit. Stud Health Technol Inform.

[33] The Insight Toolkit Library. http://www.itk.org. Accessed 26 May 2016.

[34] Desikan RS, Ségonne F, Fischl B, Quinn BT, Dickerson BC, Blacker D, Buckner RL, Dale AM, Maguire RP, Hyman BT, Albert MS, Killiany RJ (2006). An automated labeling system for subdividing the human cerebral cortex on MRI scans into gyral based regions of interest. NeuroImage.

[35] Fischl B (2012). FreeSurfer. NeuroImage.

[36] Cardinale F, Chinnici G, Bramerio M, Mai R, Sartori I, Cossu M, Lo Russo G, Castana L, Colombo N, Caborni C, De Momi E, Ferrigno G (2014). Validation of freesurfer-estimated brain cortical thickness: comparison with histologic measurements. Neuroinformatics.

[37] Rosas HD, Liu AK, Hersch S, Glessner M, Ferrante RJ, Salat DH, van der Kouwe A, Jenkins BG, Dale AM, Fischl B (2002). Regional and progressive thinning of the cortical ribbon in Huntington’s disease. Neurology.

[38] Cardinale F, Pero G, Quilici L, Piano M, Colombo P, Moscato A, Castana L, Casaceli G, Fuschillo D, Gennari L, Cenzato M, Lo Russo G, Cossu M (2015). Cerebral angiography for multimodal surgical planning in epilepsy surgery: description of a new three-dimensional technique and literature review. World Neurosurg.

[39] Avanzini P, Abdollahi RO, Sartori I, Caruana F, Pelliccia V, Casaceli G, Mai R, Lo Russo G, Rizzolatti G, Orban GA (2016). Four-dimensional maps of the human somatosensory system. Proc Natl Acad Sci USA.

[40] Liava A, Mai R, Cardinale F, Tassi L, Casaceli G, Gozzo F, Cossu M, Nobili L, Castana L, Sartori I, Lo Russo G, Francione S (2016). Epilepsy surgery in the posterior part of the brain. Epilepsy Behav.

[41] Princich J, Wassermann D, Latini F, Oddo S, Blenkmann A, Seifer G, Kochen S (2013). Rapid and efficient localization of depth electrodes and cortical labeling using free and open source medical software in epilepsy surgery candidates. Front Neurosci.

